# Demographic profile, clinical features, and outcome of peripapillary subretinal hemorrhage: an observational study

**DOI:** 10.1186/s12886-020-01426-9

**Published:** 2020-04-19

**Authors:** Ming Zou, Yi Zhang, Xi Huang, Sheng Gao, Junjun Zhang

**Affiliations:** Department of Ophthalmology, West China Hospital, Sichuan University, No. 37 Guoxue Lane, Chengdu, 610041 Sichuan China

**Keywords:** Intrapapillary hemorrhage, Myopia, Peripapillary subretinal hemorrhage, Valsalva retinopathy

## Abstract

**Background:**

To evaluate the etiology, demographic profile, clinical features, and outcomes in patients with peripapillary subretinal hemorrhage (PSH).

**Methods:**

Thirty-eight eyes of 37 consecutive patients with PSH were enrolled in this prospective observational study over 4 years; all were followed for 2 years. The main outcome measures were demographic profile, possible etiology, clinical features, outcome, and prognosis.

**Results:**

Sixty-eight percent (26/38) of eyes were in female patients; the mean patient age was 20 years. Only 1 patient (1/37) showed bilateral involvement. All patients experienced acute onset of PSH. All eyes were myopic and their best-corrected visual acuities ranged from 20/1000 to 20/12.5. The fundus features of affected eyes were classified into 3 groups: (1) PSH alone (4/38 eyes, 10.5%); (2) PSH with intrapapillary hemorrhage (17/38 eyes, 44.7%); (3) PSH with intrapapillary and vitreous hemorrhage (17/38 eyes, 44.7%). PSH occurred in nasal edges of optic discs with a crescent shape and dull-red color. All affected optic discs were small and crowded, exhibiting variable degrees of tilting. The cup of affected optic discs was narrower and deeper than that of normal control discs. Other ancillary tests provided no additional value. After a mean follow-up of 2.85 months, the hemorrhages resolved spontaneously without sequelae. Recurrence of disease was not observed in any patients.

**Conclusions:**

PSH is common in myopic eyes with tilted optic discs. We suspect that these hemorrhages occurred as a result of abrupt movement acting on a morphologically vulnerable optic disc.

## Background

A clinical syndrome of peripapillary subretinal hemorrhage (PSH) with intrapapillary hemorrhage (IPH) has been reported by Cibis [1], Kokame [2, 3], Katz and Hoyt [4], and Sibony [5]. It is described as a benign condition characterized by (1) acute onset, (2) IPH, (3) adjacent PSH, (4) increased frequency in myopic eyes, and (5) spontaneous resolution without sequelae [1–5].

Most cases have been reported in healthy young individuals of Asian ethnicities [3, 4, 6, 7]. The cause of this syndrome is uncertain, although vitreous traction on the disc [1, 3–5, 8], acute disc edema [3], and Valsalva maneuvers [3, 6, 7] have been proposed as possible causes. This prospective study was performed to describe the clinical features and analyze possible etiologies of PSH.

## Methods

Thirty-seven consecutive patients with isolated PSH, with or without IPH at presentation, were identified and evaluated from July 2012 through June 2016 in the Department of Ophthalmology, West China Hospital, Sichuan University, Chengdu, China. We excluded patients with optic disc edema, papillitis, ischemic optic neuropathy, optic nerve head drusen, juxtapapillary neovascular membrane, or glaucoma with optic disc hemorrhage. Patients taking corticosteroids or traditional Chinese herbs to relieve hemorrhage were also excluded.

Medical and ocular history, ocular examination, and ancillary tests were meticulously performed for each patient. All patients underwent best-corrected visual acuity (BCVA) assessment, slit-lamp examination, dilated fundus evaluation with indirect ophthalmoscopy or with 90-diopter lens on slit-lamp, fundus photography (FP, Zeiss FF450^plus^, Carl Zeiss Meditec AG, Jena, Germany), optical coherence tomography (OCT, Spectralis HRA-OCT, Heidelberg Engineering, Heidelberg, Germany), and fundus fluorescence angiography (FFA, Zeiss FF450^plus^, Carl Zeiss Meditec AG). For differential diagnosis, other ancillary tests were performed in selected cases at the time of initial presentation; these tests included visual field test, B-scan ultrasonography, and auto-fluorescence of fundus (AF). B-scan ultrasonography (17 eyes) was performed to exclude drusen and to assess vitreous attachment to the disc. Fourteen eyes underwent visual field testing to exclude optic disc neuropathy. Six eyes underwent AF tests to differentiate retinal appearance from that of optic nerve head drusen. Three patients underwent computed tomography (CT) and 1 patient underwent magnetic resonance imaging (MRI) for head pathology.

No treatment was given to the patients; they were closely observed throughout the study period. Monthly follow-up was performed until the hemorrhage had completely resolved. The visit at which hemorrhage absorption was complete was defined as Visit X. If X < 6 months from the time of presentation, the next visit was at 6 months post-presentation; if X ≥ 6 months, the next visit was at 12 months post-presentation. The total period of follow-up was 2 years. Detailed follow-up criteria are shown in Table 1.

**Table 1:** Follow-up Criteria

	Visit 0	Visit 1	Visit 2	Visit 3…	Visit X*	Visit X + 1	Visit X + 2	End-point
Time	Presentation	1 month	2 months	3 months…	X months	6 months	12 months	24 months
BCVA	✖	✖	✖	✖	✖	✖	✖	✖
Slit-lamp examination	✖	✖	✖	✖	✖	✖	✖	✖
FP	✖	✖	✖	✖	✖	✖	✖	✖
FFA	✖							
OCT	✖				✖			✖
Ancillary tests	✖							

*BCVA* best-corrected visual acuity, *FP* fundus photography, *FFA* fundus fluorescence angiography. Ancillary tests included visual field test, B-scan ultrasonography, auto-fluorescence of fundus (AF), computed tomography (CT), and magnetic resonance imaging (MRI)

Visit X*: Monthly follow-up was performed until the hemorrhage had completely resolved. The visit at which hemorrhage absorption was complete was defined as Visit X. If X < 6 months from the time of presentation, the next visit was at 6 months post-presentation (Visit X + 1); if X ≥ 6 months from the time of presentation, the next visit was 12 months post-presentation (Visit X + 2)

The major outcome measures included BCVA, the duration of hemorrhage relief, the appearance of hemorrhage, appearance of the optic disc, and morphology of the optic disc in OCT.

### Statistical analysis

Statistical hypothesis test and *p* values comparing means were determined using one-way analysis of variance and Post Hoc multiple comparisons. The paired *t*-test was used to determine the difference of BCVA between the first and the endpoint visit.

## Results

**Fig. 1:**
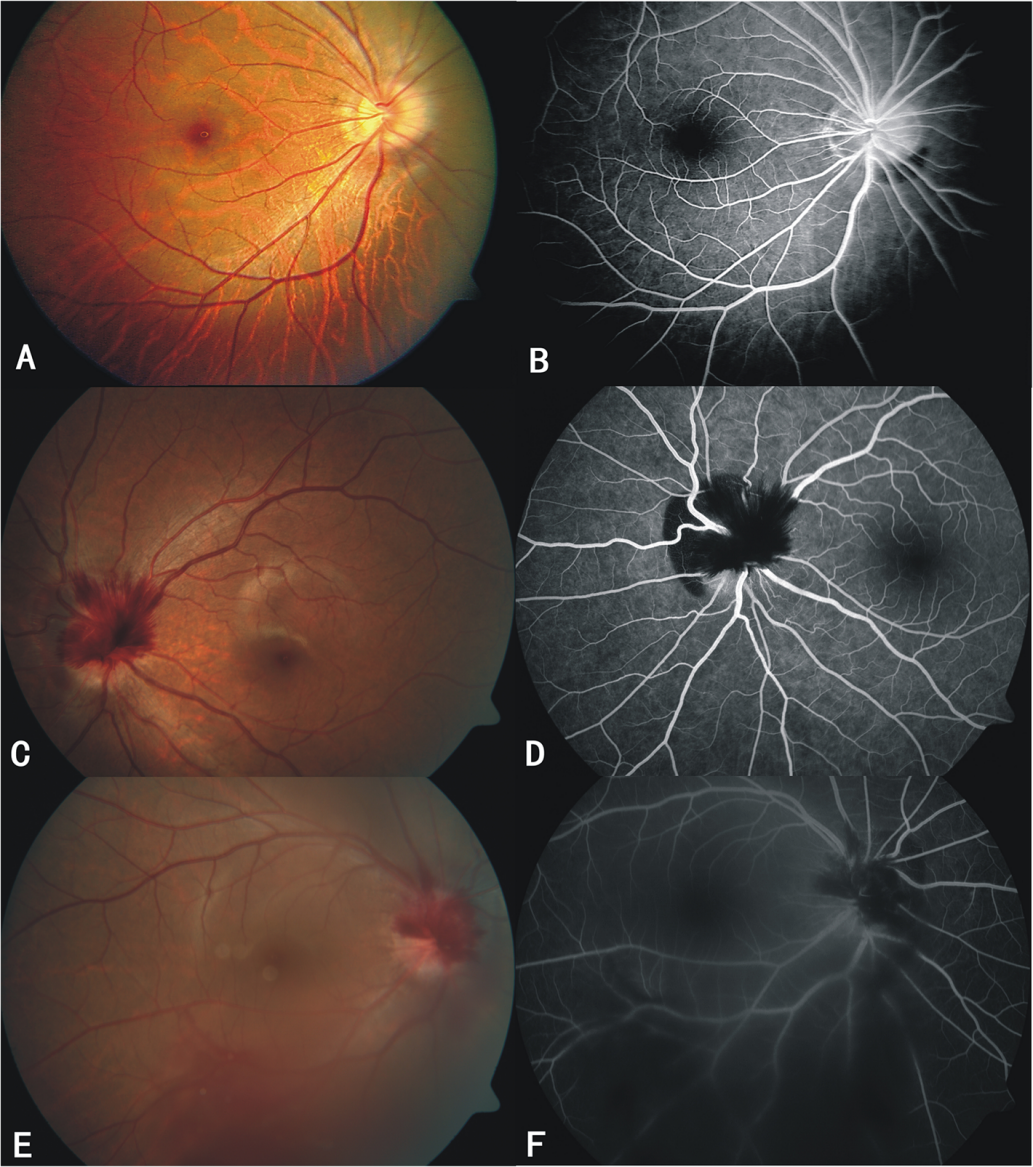
Fundus photography (FP) and fundus fluorescein angiography (FFA) showing hemorrhages classified into 3 groups. Group 1 (**a**, **b**): peripapillary subretinal hemorrhage (PSH) alone; Group 2 (**c**, **d**): PSH accompanied intrapapillary hemorrhage; Group 3 (**e**, **f**): PSH, intrapapillary hemorrhage accompanied by vitreous bleeding. FFA and FP showing PSH located subretinal space in the nasal edge of optic disc, intrapapillary hemorrhage located in the optic disc superficially, and vitreous bleeding located around the optic disc and in the inferior vitreous cavity

Our case series included 37 consecutive patients with 38 affected eyes (Table 2). There were 26 females (70.3%) and 11 males (29.7%). Thirty-six patients had unilateral presentation and 1 had bilateral involvement. Patient age ranged from 10 to 39 years (mean, 20.05 ± 7.47 years). According to the dilated fundus evaluation and FP, the hemorrhage features were as below: all manifestations of PSH were crescent-shaped, dull-red, and located in the nasal area of the optic disc with a distinct boundary. PSH was never located in the temporal area of the optic disc. IPH was superficial and radiated from the surface of the optic disc. The center of radial bleeding was clearly located in the center of the optic disc. Vitreous hemorrhage always occurred in the region of the optic disc or in the inferior cavity, due to gravity. PSH was present in 38 eyes (100%), IPH was present in 34 eyes (89.5%), and vitreous hemorrhage was present in 17 eyes (44.7%). There were no instances of IPH alone or IPH combined with vitreous hemorrhage that did not also exhibit PSH. We defined the fundus performance into 3 groups (Fig. 1): Group 1, PSH alone (4 eyes, 10.5%); Group 2, PSH with IPH (17 eyes, 44.7%); Group 3, PSH with IPH combined with vitreous hemorrhage (17 eyes, 44.7%). Notably, there were no significant differences among these 3 groups with respect to age (Table 2).

**Table 2:** Subject demographics and clinical characteristics

	Group 1	Group 2	Group 3	Total	*p*^*#*^ value	
	PSH	PSH + IPH	PSH + IPH + VH			
	(*n*^a^ = 4)	(*n* = 17)	(*n* = 17)	(*n* = 38)		
Age (years)						
Mean ± SD	18.75 ± 7.50	22.12 ± 8.89	18.29 ± 5.60	20.05 ± 7.47	*ptotal*	0.315
Range	15–30	10–39	11–31	10–39		
Gender, N (%)						
Male	3 (75%)	2 (11.8%)	7 (41.2%)	12 (31.6%)^b^		
Female	1 (25%)	15 (88.2%)	10 (58.8%)	26 (68.4%)		
Risk factor	1	3	6	10		
Male	1	1	2	4^c^		
Female	0	2	4	6		
Myopia (Diopters)						
Mean ± SD	2.88 ± 1.09	3.28 ± 1.39	3.57 ± 2.24	3.37 ± 1.77	*ptotal*	0.758
Range	2.00–4.25	1.75–7.00	1.25–9.00	1.25–9.00		
BCVA (LogMAR)						
Visit 0 (Mean ± SD)	−0.02 ± 0.11	0.13 ± 0.17	0.46 ± 0.50	0.26 ± 0.39	*ptotal*	0.012
					*p12*	*0.16*
					*p13*	*0.01*
					*p23*	*0.05*
Endpoint (Mean ± SD)	−0.06 ± 0.08	−0.03 ± 0.09	0.01 ± 0.06	−0.02 ± 0.08	*ptotal*	0.189
Duration of absorption (months)						
Mean ± SD	2.00 ± 0.82	2.53 ± 1.07	3.59 ± 1.42	2.95 ± 1.33	*ptotal*	0.018
					*p13 < 0.05*	
					*p12 > 0.05*	
					*p23 > 0.05*	
Range	1–3	1–4	2–6	1–6		
Recurrence	0	0	0	0		

*BCVA* best-corrected visual acuity, *IPH* intrapapillary hemorrhage, *PSH* peripapillary subretinal hemorrhage, *SD* standard deviation, *VH* vitreous hemorrhage

^#^: *ptotal*: *p* value of comparison among all groups; *p12*: *p* value of comparison between Group 1 and Group 2; *p13*: *p* value of comparison between Group 1 and Group 3; *p23*: *p* value of comparison between Group 2 and Group 3

^a^Affected eyes

^b^One male both eyes affected

^c^Bilateral eyes affected man, his right eye in Group 1, his left eye in Group 3

All patients exhibited an acute onset at first presentation, in which they complained of blurred vision, floaters, smudges in the visual field, and reduced visual acuity. None had significant prior medical problems or signs of trauma. Tracing the illness of history, 1 female patient had experienced vomiting, 1 male patient had experienced coughing, 2 female patients had experienced constipation, 1 male patient had performed broad jumping (a sport), and 4 patients (3 females and 1 male) had felt anxiety due to final examinations in high school. The male patient who had performed broad jumping exhibited bilateral involvement. We interpreted these events as risk factors (Table 2). Other patients had no identifiable causes.

BCVAs at initial presentation ranged from 20/1000 (Logarithm of the minimal angle of resolution, LogMAR 1.70) to 20/12.5 (LogMAR − 0.20) (Table 2). There was no ocular dysfunction prior to the onset of PSH. The mean LogMAR BCVAs in Groups 1, 2, and 3 were − 0.02, 0.13, and 0.46, respectively. Mean BCVA in Group 3, in which patients exhibited vitreous hemorrhage, was worse than that in the other 2 groups, in which patients did not exhibit vitreous hemorrhage (*p13* = 0.006, *p23* = 0.047; Table 2). At the end of follow-up, mean BCVAs for all groups were normal, such that there was no significant difference among the 3 groups (*p* > 0.05). Statistical analysis of BCVAs between baseline and final visit for each group had been done separately. BCVAs in Group 2 and Group 3 showed significant improvement in vision (*p <* 0.05). Hemorrhages resolved spontaneously within 1 to 6 months (mean 2.95 ± 1.33 months). The mean durations of hemorrhage absorption in Groups 1, 2, and 3 were 2.00 ± 0.82 months, 2.53 ± 1.07 months, and 3.59 ± 1.42 months, respectively. In Group 3, which had vitreous hemorrhage, the duration of bleeding relief was longer than in Group 1 and no difference with Group 2 (*p13 < 0.05, p23 > 0.05*; Table 2). There were no sequelae and no recurrences during follow-up.

All patients had myopia with a mean spherical equivalent of 3.37 ± 1.77 diopters (Table 2). Four eyes had high myopia (spherical equivalent > 6.0 diopters), 17 eyes had moderate myopia (spherical equivalents of 3.0–5.75 diopters) and 17 eyes had mild correction (spherical equivalents of 0.25–2.75 diopters). The mean spherical equivalents were not significantly different among the groups (*p* > 0.05; Table 2).

FFA was performed in all patients at the time of initial presentation. Angiograms showed blocked hypofluorescence from hemorrhage and mild late leakage of fluorescence located at the margin of the optic disc. There were no hyperfluorescence signs indicative of neovascularization or any other abnormal conditions. B-scan in 17/37 patients and OCT in all patients showed no evidence of drusen or vitreous traction at the optic nerve head. Visual field tests were performed in 14 eyes; 10 were normal and 4 exhibited enlargement of the physiologic scotoma, although this had no specific clinical value. All CT (performed in 3/37 patients) and MRI (performed in 1/37 patients) outcomes were normal.

**Fig. 2:**
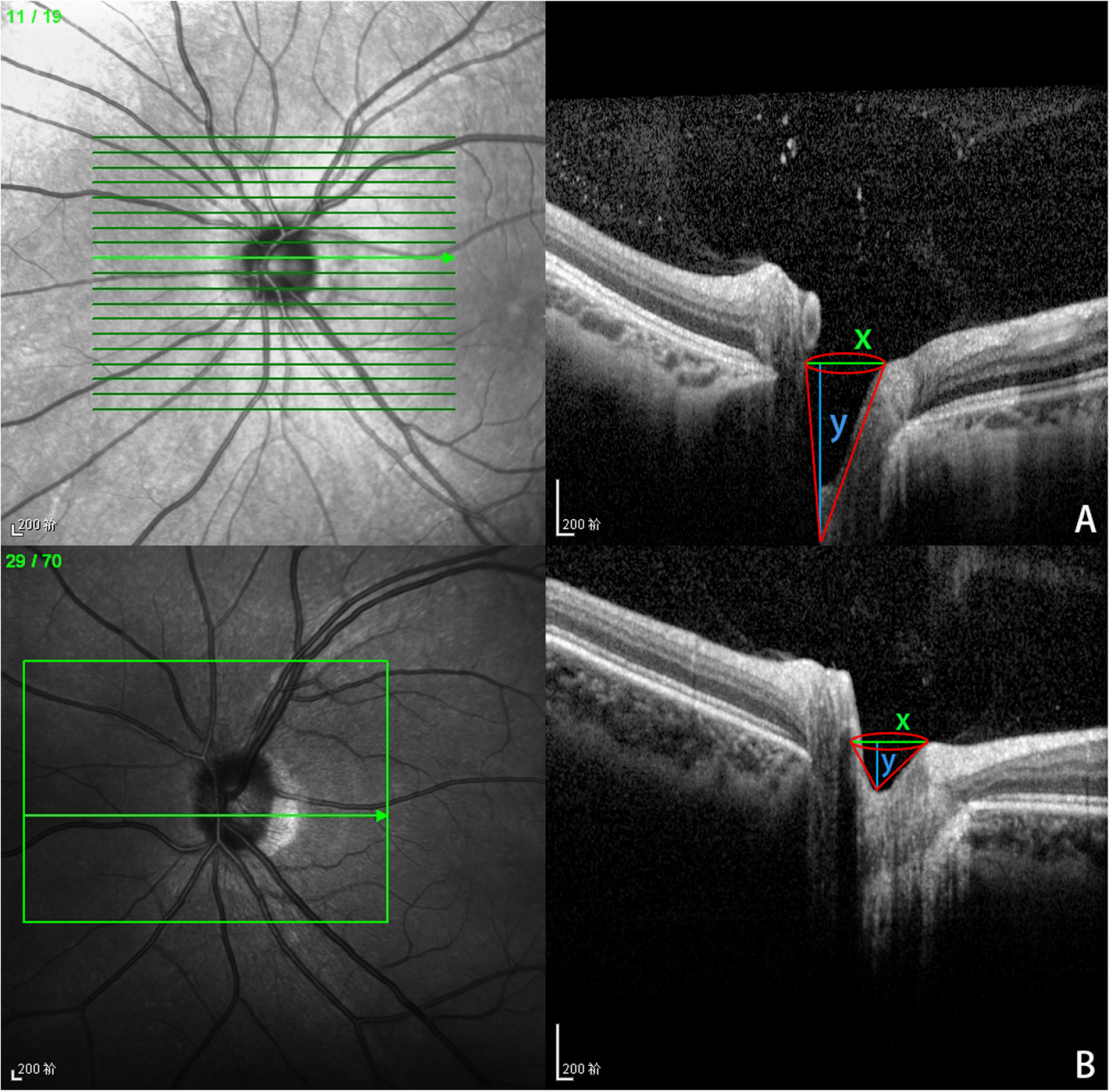
Optical coherence tomography (OCT) images: showing affected optic disc (**a**) exhibits sharp insertion of nerve fiber at the nasal region, and the cup is deep and narrow compare to the normal control (**b**). The shape of the optic cup is outlined with a cone (in red). The diameter (x, in green) at the bottom of the cone represents the opening size of the optic cup, and the height (y, in blue) of the cone represents the depth of the cup. The smaller the x value is, and the larger the y value is, which means that the optic disc is narrower and deeper

When we analyzed the shapes of the optic disc and PSH, we found that they were obstructed by regions of hemorrhage in 25 eyes at the time of initial presentation. The optic discs of these patients were evaluated at the visit which hemorrhage absorption was complete. OCT images (Fig. 2) showed that all affected optic discs were small. They exhibited variable degrees of oblique insertion, accompanied by elevation and blurring of the nasal regions of the nerve head. The cup of affected optic discs, compared with that of normal control optic discs, was much narrower and deeper. Moreover, the insertion angle of the nerve fiber in the nasal region was sharper. We outlined the shape of the optic cup using a cone. The diameter (x) at the bottom of the cone represented the opening size of the optic cup, and the height (y) of the cone represented the depth of the cup. The smaller the x value was, the larger the y value was, which means that the optic disc was narrower and deeper. We analyzed the cone shape of all subjects, among which the y value of 73.7% (28/38) eyes was significantly higher than the x value. Under normal circumstances, the difference between x value and y value was not significant. Importantly, the regions exhibiting PSH and optic disc elevation were consistent with each other. Optic disc features were highly related to the cause of hemorrhage.

## Discussion

This clinical syndrome of PSH with or without IPH was described in 1975 [1]. It has been observed in people with European ethnicity [5], but is most common in people with Asian ethnicities [3, 4, 6, 7]. In our study, the syndrome comprised a benign condition with acute onset, which spontaneously resolved over a period of several months without sequelae or recurrence. Notably, this syndrome is more common in myopic eyes and in younger people. The characteristics of our case series are similar to those of other published reports [1–7].

There had no effect on visual acuity if PSH was present alone, because the BCVAs in Group 1 had no difference between the baseline and final visit. There was symptomatic when retina exhibited IPH brought out thickening of retina. There had mild to moderate decline of visual acuity depending on the amount of hemorrhages, especially the vitreous hemorrhages occurred. With the absorption of bleeding, vision improved. It also confirmed that there was no neurological insult to the nerve, otherwise the vision would not be recovered fully due to absorption of bleeding. The patients in Group 1 had normal vision but symptoms. This might be due to the presence of IPH at the onset of PSH. When they came to the clinic, IPH had absorbed already. In other words, patients in different groups might be in different clinical stages not different classifications.

In prior studies, IPH has been considered more influential with respect to the outcome, relative to PSH and vitreous hemorrhage [1–4, 7]. Accordingly, the most commonly proposed cause has been acute vitreopapillary traction [1, 3–5, 8], which induced superficial bleeding of the optic disc. In our study, PSH was present in 38 eyes (100%), however IPH was present in 89.5% (34 eyes). According to FFA and FP results, PSH alone (without IPH, Group 1) was found in 4 eyes (10.5%). In contrast, IPH without PSH was not found. All clinical characteristics suggested that PSH was the more basic aspect of bleeding in our case series. Thus, we consider PSH to be the fundamental manifestation.

The vascular anatomy of the optic nerve head has a unique structure, and its vascular supply varies with the region [9–12]. The superficial nerve fiber layer (NFL) is principally supplied by arterioles in the adjacent retina. Most of these vessels are capillaries that originate from peripapillary branches of the retinal arteries. The prelaminar portion receives its arterial supply via direct branches of the short posterior ciliary arteries, as well as vessels originating from the circle of Zinn-Haller. Branches of the short posterior ciliary arteries may pass through the choroid. The circle of Zinn-Haller communicates with the peripapillary retinal arterioles. The venous system almost exclusively drains to the central retinal vein, with minor contributions to peripapillary choroidal veins. Based on the morphological features of bleeding, PSH may originate from capillaries of choroidal origin, whereas IPH may originate from capillaries of retinal origin in the NFL. Because PSH is the primary manifestation of this syndrome, the deep part of the retina or choroid might be the original site of bleeding. Depending on the extent of involvement, triggering events cause choroidal vascular bleeding, and develop into retinal vascular bleeding, which has a tendency from deep to superficial. There is no sign of vitreopapillary traction in B-ultrasound and OCT [2, 3, 5]. This observation confirms that bleeding does not originate superficially, primarily from deep part of the optic disc.

The cause of acute onset of hemorrhage is not certain [3–5]. Existing theories suggest the involvement of acute vitreopapillary traction [1, 4, 5, 8] and acute disc edema [3]. Some studies have reported evidence of vitreopapillary traction based on B-scan ultrasonography and OCT [1, 4]. However, vitreoretinal relationships were not confirmed in other series [3, 5]. In our study, there was no evidence for vitreous traction based on B-scan ultrasonography and OCT. In addition, vitreous traction induces superficial IPH, which is incompatible with the observation of eyes with PSH alone. Based on FFA results, Kokame et al. [3] postulated that acute disc edema may be the cause of PSH. In prior studies, FFA has shown hyperfluorescence of disc staining [2, 3, 5]. Kokame et al. suggested that acute disc edema was due to inflammation [3]. However, in our study, there was no apparent fluorescence leakage of the papillary vascular network, nor was there was direct evidence of inflammation. Furthermore, 4 patients in the current series were found no specific signs in the brain and orbit (3 underwent head CT and 1 underwent MRI). These results suggest that there may be other acute triggers of hemorrhage.

The Valsalva maneuver was considered as another possible cause in previous studies [3, 7]. Sneezing [3, 13] was observed as a cause of bleeding in a prior study. The authors postulated that the Valsalva maneuver places a shearing force on the vulnerable capillaries of choroidal origin on the nasal edge of the disc [3, 13]. Moreover, the Valsalva maneuver may increase the pressure of the venous system [14, 15]. Because of the sudden increase in venous pressure, retinal capillaries spontaneously rupture [14–19]. Notably, retinal capillaries supply the NFL layer [11]; thus, this rupture manifests as IPH. In addition, the classical pattern of Valsalva retinopathy often presents as preretinal hemorrhage or sub-internal limiting membrane hemorrhage [16–19]. It demonstrates that the original bleeding site in cases caused by the Valsalva maneuver is of superficial retinal origin. In our study, only 10 eyes (10/38) showed evidence of the Valsalva maneuver. Thus, we consider the Valsalva maneuver to be a possible cause for intrapapillary bleeding in our series, but not a cause of PSH.

Tilted optic discs have generally been regarded as a risk factor for bleeding in prior studies [2–5, 7, 9], similar to the findings in our study. These discs appear small, crowded, and elevated in the nasal regions. The insertion angle of the nerve fiber in the nasal region is sharp, and the cup of affected optic discs is narrow and deep. These unique morphological features might draw the retinal and choroidal tissues over and around the elevated region. The vascular network of affected optic discs is tightened and exhibits high tension, which makes it vulnerable to bleeding. We speculate that capillaries of choroidal origin in the nasal region are most susceptible to bleeding with sufficient irritation.

Based on our findings, we suspect that a sudden fast movement may be the trigger to initiate bleeding in patients with PSH. First, when rapid eye movement occurs, the retinal and choroidal tissue with high tension in the elevated region might be strongly pulled in 1 particular direction. In this situation, susceptible deep retinal and choroid tissues are more likely to bleed. Second, depending on the extent of bleeding and the distribution of triggered vessels, blood may drain into the vitreous cavity. When the vascular supply for NFL is affected, IPH appears. Finally, this movement occurs abruptly, where 1 eye moves in 1 particular direction and causes the susceptible retinal tissue to bleed; however, the other eye, which may exhibit the same unique morphologic structure, moves in the opposite manner (with respect to orientation within the socket and the muscles that cause movement) and may even relax. This might explain why the bleeding is typically observed unilaterally.

Our study has several limitations. First, the study designed without intervention is drawback. Second, although we performed study procedures on the first day of clinical visit, subjects in the study required extra time in or an extra trip to the clinic, not the initial time of onset, which may present bias on the clinical manifestation of inclusion subjects. Another potential limitation is that there were no control subjects. Considering the fact that anatomical peculiarity of optic discs is the basis, quantification from OCT and comparison with that of normal discs warranted further study.

## Conclusions

PSH syndrome is more common in younger patients with myopic eyes that exhibit tilted optic discs. It presents with variable clinical manifestations, such as PSH alone, PSH with IPH, and PSH with intrapapillary bleeding accompanying vitreous hemorrhage. Notably, PSH is not a single entity. We postulate that its causes may include sudden movement of myopic eyes with elevated optic discs. It is easy to misdiagnose PSH as optic disc edema, papillitis, buried optic nerve drusen, or other phenomena. The challenge for clinicians is to recognize the characteristics of PSH and distinguish this condition from others with similar features.

## Data Availability

The datasets used and/or analyzed during the current study are available from the corresponding author on reasonable request.

## Abbreviations

AF: Auto-fluorescence of fundus
BCVA: Best-corrected visual acuity
CT: Computed tomography
FFA: Fundus fluorescence angiography
FP: Fundus photography
IPH: Intrapapillary hemorrhage
LogMAR: Logarithm of the minimal angle of resolution
MRI: Magnetic resonance imaging
NFL: Nerve fiber layer
OCT: Optical coherence tomography
PSH: Peripapillary subretinal hemorrhage
